# Pilot Study: Estimation of Stroke Volume and Cardiac Output from Pulse Wave Velocity

**DOI:** 10.1371/journal.pone.0169853

**Published:** 2017-01-06

**Authors:** Yurie Obata, Maki Mizogami, Daniel Nyhan, Dan E. Berkowitz, Jochen Steppan, Viachaslau Barodka

**Affiliations:** 1 Division of Cardiac Anesthesia, Department of Anesthesiology and Critical Care Medicine, The Johns Hopkins University School of Medicine, Baltimore, MD, United States of America; 2 Department of Anesthesiology and Reanimatology, University of Fukui, Fukui, Japan; Kurume University School of Medicine, JAPAN

## Abstract

**Background:**

Transesophageal echocardiography (TEE) is increasingly replacing thermodilution pulmonary artery catheters to assess hemodynamics in patients at high risk for cardiovascular morbidity. However, one of the drawbacks of TEE compared to pulmonary artery catheters is the inability to measure real time stroke volume (SV) and cardiac output (CO) continuously. The aim of the present proof of concept study was to validate a novel method of SV estimation, based on pulse wave velocity (PWV) in patients undergoing cardiac surgery.

**Methods:**

This is a retrospective observational study. We measured pulse transit time by superimposing the radial arterial waveform onto the continuous wave Doppler waveform of the left ventricular outflow tract, and calculated SV (SV_PWV_) using the transformed Bramwell-Hill equation. The SV measured by TEE (SV_TEE_) was used as a reference.

**Results:**

A total of 190 paired SV were measured from 28 patients. A strong correlation was observed between SV_PWV_ and SV_TEE_ with the coefficient of determination (R^2^) of 0.71. A mean difference between the two (bias) was 3.70 ml with the limits of agreement ranging from -20.33 to 27.73 ml and a percentage error of 27.4% based on a Bland-Altman analysis. The concordance rate of two methods was 85.0% based on a four-quadrant plot. The angular concordance rate was 85.9% with radial limits of agreement (the radial sector that contained 95% of the data points) of ± 41.5 degrees based on a polar plot.

**Conclusions:**

PWV based SV estimation yields reasonable agreement with SV measured by TEE. Further studies are required to assess its utility in different clinical situations.

## Introduction

In clinical practice, hemodynamic monitoring during anesthesia and in intensive care units is of paramount importance. The goal of hemodynamic monitoring is to guide interventions as well as to ensure adequate end organ perfusion and oxygen delivery by optimizing stroke volume (SV) and cardiac output (CO). Conventional hemodynamic variables such as arterial blood pressure, central venous pressure, and urine output are frequently used as surrogates for adequate end organ perfusion.[1] However, these parameters are not able to directly measure SV and hence CO. Although the pulmonary artery catheter (PAC) is still considered the gold standard to monitor SV and CO, its invasive nature and potential for life-threatening complications largely restrict its use in the modern era.[2][3] Recently, multiple non or minimally invasive SV/CO monitoring devices were introduced into clinical practice, such as pulse contour analysis devices, esophageal Doppler devices, the partial carbon dioxide rebreathing technique, and transthoracic electrical bioimpedance measurements. However, they have not yet replaced PACs due to poor trending ability and inadequate agreement with the clinical standard (PAC).[4] Meanwhile, echocardiography either, transesophageal (TEE) or transthoracic (TTE), has become a frequently utilized monitor, especially in the cardiac operating rooms and intensive care units. TEE based SV/CO has been validated against PAC based values with good limits of agreement.[5][6] While ultrasound based techniques have a number of advantages, they have several important limitations, such as the difficulty of continuous real time monitoring, interference from electric cautery, and operator dependence.[7] Moreover, TEE is an invasive technique and is poorly tolerated by un-sedated patients. There have been reports of continuous CO measurements based on pulse transit time, which is inexpensive and easy to use but showed poor agreement with gold standard.[8][9] To overcome these disadvantages, we focused on developing a real time, continuous, non-invasive technique to estimate SV and CO. Pulse wave velocity (PWV) is a surrogate measure of vascular properties in general and vascular stiffness in particular.[10] We hypothesize that SV and CO estimated from PWV are comparable to SV and CO derived from TEE. Hence, the aim of this present proof of concept study is to compare the accuracy and the trending ability of SV, estimated from PWV utilizing the Bramwell-Hill equation, with SV measured using TEE in patients undergoing cardiac surgery.

## Materials and Methods

### Subjects

This retrospective observational study utilized data from cardiac surgery patients who underwent surgery at The Johns Hopkins Hospital between October 2011 and September 2013. The protocol was approved by the Johns Hopkins Medicine Institutional Review Boards (IRB00088711). The requirement for written informed consent was waived by the IRB.

The study included patients, 18 years of age or older undergoing cardiac surgery who had TEE images stored in the database on which the radial arterial waveform was superimposed on the continuous wave (CW) Doppler waveform of the left ventricular outflow tract (LVOT). All images were obtained before the initiation of cardiopulmonary bypass (CPB). Values of blood pressure (BP) at the time of image acquisition was recorded simultaneously. Patients with severe aortic valve stenosis, severe aortic valve insufficiency, arrhythmias, or an implanted device for cardiac rhythm management were excluded.

### Measurements

**Fig 1:**
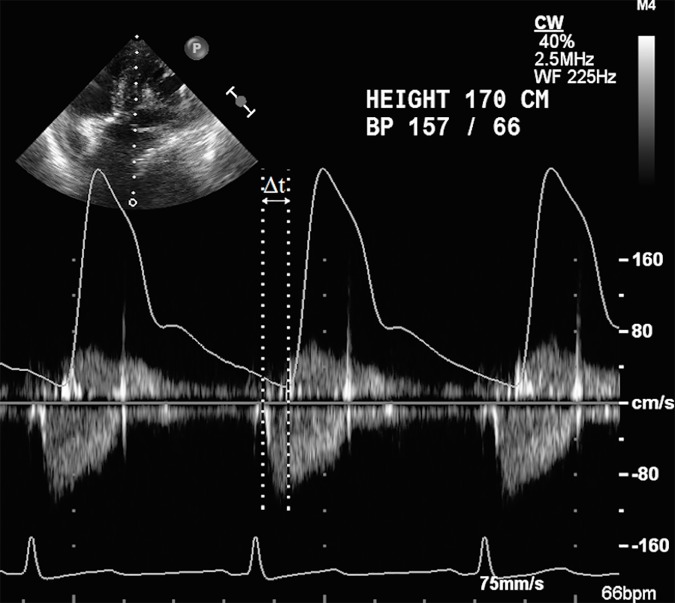
Representative image of measurements. We measured the pulse transit time (Δt) from the start of ejection on continuous wave Doppler waveform to the upstroke on the radial arterial waveform, and left ventricular outflow tract velocity time integral by tracing the continuous wave Doppler waveform.

Intraoperative care was similar in all patients, each of whom received general anesthesia with most patients receiving a combination of midazolam (2–10 mg), fentanyl (750–2000 μg), vecuronium (10–20 mg), and isoflurane (0.5–1%) after placement of a right sided 20 gauge radial arterial catheter for continuous BP measurements. A TEE probe was inserted after the induction of general anesthesia, and a comprehensive TEE examination performed by a cardiac anesthesiologist with advanced echocardiography certification using a Philips iE33 ultrasound machine (Philips Medical Systems, Amsterdam, Netherlands). The arterial pressure and electrocardiogram (ECG) waveforms were recorded and simultaneously projected onto the LVOT CW Doppler waveform by connecting the clinical monitor (GE Healthcare, Chicago, IL) to the TEE machine, at the identical speed of 75mm/s (Fig 1). The CW Doppler images were obtained from either the deep trans-gastric or the trans-gastric long axis view of the aortic valve. All images were stored on the hospital server and the offline measurements were performed using the software “Synapse Cardiovascular” (FUJIFILM, Tokyo, Japan) by two independent readers who were blinded to the intraoperative management and to the cardiac anesthesiologist who obtained the images.

Stroke volume (SV_PWV_) was calculated using the transformed Bramwell-Hill equation. The Bramwell-Hill equation was introduced in 1922 allowing the estimation of PWV from an increase in the arterial volume (dV), an increase in blood pressure (dP), the arterial tree volume (V), and blood density (ρ).[11]

PWV=√(V/[ρ×dV/dP]) (1)

We simplified dV and dP to SV and pulse pressure (PP) respectively since arterial compliance (dV/dP) is linearly related to SV/PP: PWV = √ (V / [ρ × SV / PP]).[12] Solving the equation for SV enables us to estimate SV from PWV and PP. SV (ml) = (133.32 (N/m^2^) × V (ml) × PP (mmHg)) / (ρ (kg/m^3^) × PWV^2^ (m^2^/s^2^)), where 1 mmHg is 133.32 N/m^2^ (N = kg × m/s). Arterial blood volume is estimated as 11% of the total circulating blood volume (TBV), hence V was calculated using the formula, V = TBV × 0.11.[13] TBV was calculated using Nadler’s formula with height, weight, and gender as described previously.[14] We used ideal body weight (IBW) (Males: IBW = 50 kg + 2.3 kg for each inch over 5 feet, Females: IBW = 45.5 kg + 2.3 kg for each inch over 5 feet.) for this calculation.[15] When the actual body weight was greater than 30% of the calculated IBW, IBW was replaced to adjusted body weight (ABW: ABW = IBW + 0.4 × (actual body weight–IBW)).[16][17] PP was measured from the simultaneously recorded arterial blood pressure waveform. Since blood density does not change significantly with different levels of hemoglobin, we assumed it to be a constant value of 1055 kg/m^3^. We used aortic to radial PWV, which was estimated by dividing the predicted vascular path length (a distance from the aortic valve to the site of the radial artery catheter) by pulse transit time. We assumed the vascular path length to be a demi-span (distance from sternal notch to the tip of the fingers). Demi-span was estimated from height, age, and gender as described previously.[18] We defined pulse transit time (Δt) as the time from the foot of the CW Doppler waveform (start of ejection) to the origin of the upstroke on the arterial waveform (Fig 1).

SV of the reference method (SV_TEE_) was calculated using the formula: SV = LVOT CSA × LVOT VTI, where CSA is the cross sectional area of the LVOT and VTI is the velocity-time integral across the LVOT. LVOT VTI was measured by tracing the CW Doppler waveform across the aortic valve. Calculation of the LVOT CSA was performed by measuring the LVOT diameter from the mid esophageal long axis view, assuming a circular LVOT. In cases where more than one beat per image was captured, Δt and LVOT VTI were averaged across all beats.

### Statistical analysis

We report continuous variables as mean (± standard deviation: SD) and categorical variables as proportions. All continuous variables were normally distributed. The sample size (at least 189 measurements) was calculated for an equivalence trial with a range of equivalence of ±4.5 ml (5% of a mean SV equal to 89 ml) and acceptable limits of agreement of ±27 ml (i.e. 30% of a mean SV equal to 89 ml), with a two-sided significance of 0.05 and a power of 0.80.[19][20] SV_PWV_ was compared with SV_TEE_ using simple linear regression to obtain a coefficient of determination (R^2^) and an equation defining the relation. Agreements of absolute values between the paired SV_PWV_ and SV_TEE_ were examined by Bland-Altman analysis. The bias (the mean difference between SV_TEE_ and SV_PWV_), limits of agreement, and percentage error (PE) were calculated. The percentage error was calculated using the formula, PE = 2 SD of bias / mean value of the reference method. SV_PWV_ was evaluated as interchangeable with SV_TEE_ when the PE was 30% or less.[21] Trending ability was assessed by analyzing ΔSV values on a four-quadrant plot and a polar plot analysis using the percentage changes in ΔSV (%ΔSV). ΔSV was the difference between consecutive SV measurements. We used standard exclusion zones of 15% in the four-quadrant plot and 10% in the polar plot as suggested by Critchley.[22] The use of the exclusion zone was suggested as a way to limit measurement noise when the changes are small. In the four-quadrant plot analysis the concordance rate was defined as the percentage of data points lying in the upper right or lower left quadrant after excluding the central zone. We used a concordance rate ≥ 92% as an indicator of good trending ability in the four-quadrant plot analysis as suggested by Critchley.[22] In the polar plot analysis, a mean angular bias (i.e. a mean polar angle) of within ± 5 degrees was considered to represent a good calibration. We used the radial limits of agreement (i.e. the radial zone containing 95% of the all data points) within ±30 degrees and the angular concordance rate (i.e. the percentage of data points within ±30 degrees of the radial zone) of more than 95% as an indicator of good trending ability as suggested by Critchley.[23] Inter-observer variability for SV_PWV_ was assessed by calculating the coefficient of variability (CoV = SD of repeated measures as % of their mean). We used CoV of less than 15% as an acceptable range. SigmaPlot 13.0 (Systat Software, Inc., San Jose, California, USA) was used to construct the polar plot. The rest of the analysis was performed with GraphPad Prism version 6.0 (GraphPad Software, San Diego, California, USA). Statistical significance was set at P < 0.05.

## Results

We identified 37 patients who had images of the radial arterial waveforms superimposed on the CW Doppler waveforms of the LVOT. We excluded 8 patients with severe aortic valve stenosis and 1 patient with severe aortic valve insufficiency.

A total of 190 pairs of SV data from 28 patients were included in the analysis. The mean (SD) number of SV pairs per patient was 7 (6). No arrhythmias were present in the beats analyzed. The baseline characteristics of the patients are outlined in Table 1. None of the patients required a continuous infusion of an inotropic drug or mechanical cardiopulmonary support during image acquisition.

**Table 1:** Demographic Data and Patient Characteristics.

Variable	Data
Number of patients	28
Age (years old)	
Mean (SD)	63 (15)
Gender	
Female (%)	3 (11)
Male (%)	25 (89)
Height (cm)	
Mean (SD)	175 (10)
BMI (kg/m2)	
Mean (SD)	31 (7)
Operation	
Isolated CABG (%)	21 (75)
Valve (%)	3 (11)
Others (%)	4 (14)

BMI: body mass index, CABG: coronary artery bypass grafting.

**Fig 2:**
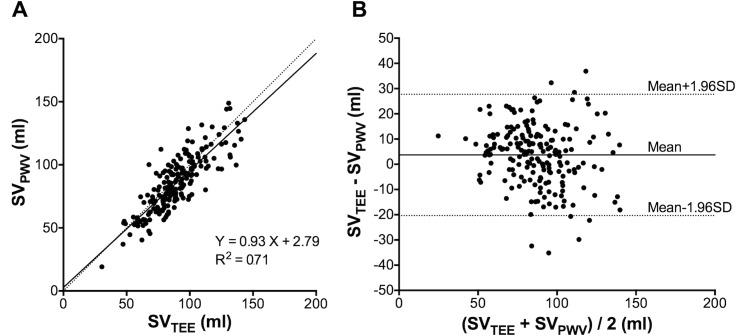
The relationship between SV_PWV_ and SV_TEE_. (A) The linear regression analysis illustrates the relationship between SV estimated from pulse wave velocity and SV measured by transesophageal echocardiography. The solid line indicates the regression, and the dashed line indicates the line of identity (x = y). (B) The Bland-Altman plots of the difference between SV_PWV_ and SV_TEE_. The solid line indicates the mean of difference (3.70 ml), and the dashed line indicates the 95% limits of agreement (from -20.33 to 27.73 ml). SV: stroke volume, SV_PWV_: stroke volume estimated from PWV, SV_TEE_: stroke volume measured by transesophageal echocardiography.

The correlation between SV_PWV_ and SV_TEE_ is shown in Fig 2A. The slope and intercept of the regression were 0.93 (95% confidence interval (CI): 0.84 to 1.01, p<0.0001) and 2.79 (95%CI: -4.96 to 10.54), respectively. The coefficient of determination (R^2^) was 0.71. A Bland-Altman analysis revealed that the bias was 3.70 ml with the limits of agreement ranging from -20.33 to 27.73 ml and a percentage error of 27.4% as presented in Fig 2B.

**Fig 3:**
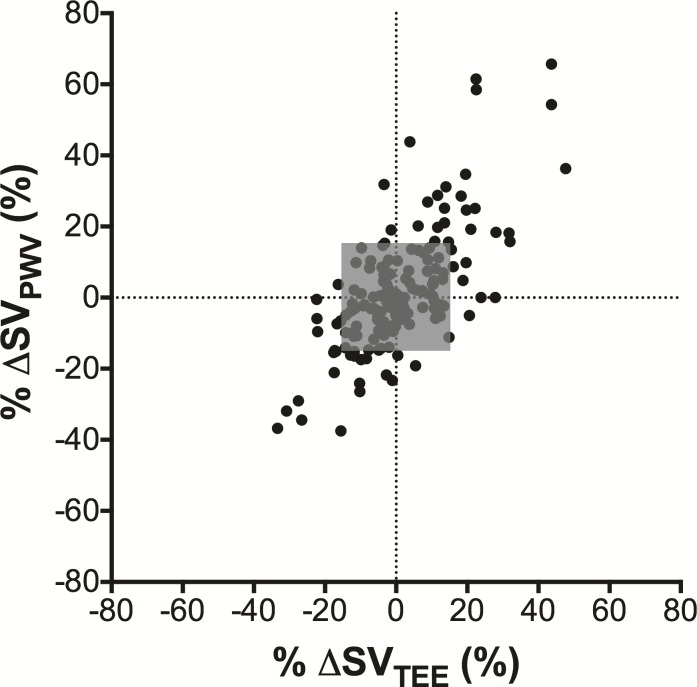
The four-quadrant plot analysis. The four-quadrant plot analysis assesses the trending ability of %SV changes as estimated from pulse wave velocity compared to %SV changes as measured by transesophageal echocardiography. The concordance rate was 85.0% with an exclusion zone of 15% (shaded grey area). SV: stroke volume, %ΔSV_PWV_: rate of change in sequential stroke volume estimated from PWV, %ΔSV_TEE_: rate of change in sequential stroke volume measured by transesophageal echocardiography.

Since analysis of the trending ability (concordance rates from four-quadrant and polar plots) requires calculation of the difference between consecutive SV measurements (ΔSV), only patients who had multiple images at different hemodynamic states could be included in this analysis. Hence 8 patients who had only one pair of SV data were excluded from the trending analysis. 162 pairs of ΔSV (%ΔSV) data were analyzed and depicted from 182 pairs of SV data from 20 patients. The four-quadrant plot for %ΔSV data is shown in Fig 3. Out of 162 depicted pairs 102 pairs were located within the 15% exclusion zone (shaded grey area). Out of 60 pairs included in the calculation of concordance rate, 51 pairs were located in either the upper right or lower left quadrant, hence the calculated concordance rate from the four-quadrant plot was 85.0% (51 / 60 × 100%).

**Fig 4:**
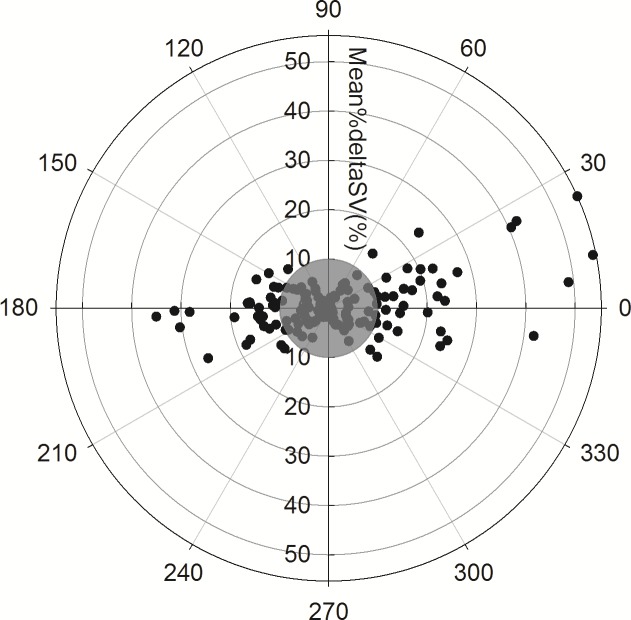
The polar plot analysis. The polar plot analysis assesses the trending ability of %SV changes as estimated from pulse wave velocity compared to %SV changes as measured by transesophageal echocardiography. The mean angular bias was 1.38 degrees with radial limits of agreement of ±41.5 degrees. The concordance rate was 85.9% with an exclusion zone of 10% (shaded grey area). SV: stroke volume, Mean%deltaSV: the mean value of sequential SV change for the reference (%ΔSV_TEE_) and test (%ΔSV_PWV_) values. The coefficient of variability (CoV: SD of repeated measures as % of their mean) to assess inter-observer variability of SV_PWV_ was 10.1%, which was within 15% of acceptable range.

The polar plot for the %ΔSV data is shown in Fig 4. Out of 162 depicted pairs, 98 pairs were located within the 10% exclusion zone (shaded grey area). The mean (SD) angular bias was 1.38 (20.73) degrees with the radial limits of agreement of ±41.5 degrees. Out of 64 pairs included in the calculation of angular concordance rate, 55 pairs were located within ±30 degrees, hence the calculated concordance rate for the polar data points was 85.9% (55 / 64 × 100%).

## Discussion

The most important finding of our proof of concept study is that SV as estimated from PWV is in good agreement with SV measured by TEE in patients undergoing cardiac surgery. Our PWV based SV calculation produced a very small and clinically insignificant bias (3.70ml) with a percentage error of 27.4%, indicating that the agreements of absolute values between SV_PWV_ and SV_TEE_ are clinically acceptable. Critchley et al. suggested using percentage errors of 30% or less between a novel technique and the current gold standard as clinically acceptable for showing that a new technique is clinically comparable to the gold standard. This percentage error of ± 30% arises from the assumption that the commonly used reference technique has a precision of ± 20% or less. The combination of two precisions of ± 20% equates to a total error of ± 28.3% (√[(±20)^2^ + (±20)^2^]), which is commonly rounded up to ± 30%.[24] In order to accept the new technology the level of accuracy and precision should at the very least be equal that of the reference technique.^21^ TEE is now widely accepted as a reliable minimally invasive method for SV and CO measurements with a reported precision of 15.6% and has been validated in comparison with the thermodilution method which has 20% precision.[5][25] Therefore, we were assured that the SV measured using TEE Doppler was appropriate as a reference method in our study. As suggested by Cecconi we used the same 30% percentage error threshold to compare SV_PWV_ to SV_TEE_ in this study.

It has been suggested that the reliable real time tracking of changes in CO is more important than the ability to deliver a highly accurate single measurement.[4][26] To assess the trending ability of different technologies, the four-quadrant and polar plot analysis have been developed. The initial studies of Critchley used the thermodilution method as a reference and have reported concordances rate of 92% with an exclusion zone of 15% (four-quadrant plot analysis) and 95% with an exclusion zone of 10% (polar plot analysis), and radial limits of agreement of within ± 30 degrees as threshold values of good trending ability.[23] Since no other threshold values have been reported, the threshold values proposed by Critchley have become the only ones used to compare methods for CO estimation regardless of the technique used. However, there are no universally accepted thresholds to describe the trending ability for studies that use TEE as a reference.[27] In our tests of trending ability, PWV based SV estimation showed promising results reaching a concordance rate of 85.0% in the four-quadrant plot analysis and radial limits of agreement of ±41.5 degrees in the polar plot analysis when compared to a TEE based SV estimation. Trending analysis is most robust when there are significant changes present in the measured parameter. In our study the majority of data points for trending analysis were within the exclusion zone indicating that there were only small variations in SV and CO. Out of the initially available 190 pairs of SV data only 60 were used in the four quadrant concordance rate calculation and only 64 in the polar plot concordance rate calculation. That might explain why our method of SV calculation did not reach the suggested thresholds for trending ability.

Our present study serves, as a proof of principle to determine if it is feasible to accurately estimate SV from PWV–using invasive monitors. Clinically, one can obtain pulse transit time and PWV by using only noninvasive or minimally invasive techniques.[28] Pulse transit time can be estimated from the peak of the R wave on ECG or from the beginning of the S1 sound of phonocardiogram to the initiation of the upstroke on the radial arterial tonometry waveform (or the plethysmograph waveform). Hence the major potential benefit of our method as a clinical monitor of CO is its ability to estimate SV and CO in a noninvasive continuous way. Moreover this technique has a strong potential to being automated by real time computerized signal analysis of the ECG, phonocardiogram, and pulse plethysmogram waveforms–which would make it non-invasive and continuous.

There are several prior studies comparing SV measured by TEE to SV measured by minimally invasive devices. Castro et al compared SVs measured by the axillary calibrated artery pulse-contour method (PiCCO monitor, Pulsion Medical Systems, Munich, Germany) to SVs measured by TEE Doppler in patients undergoing aortic surgery.[29] The percentage error of both minimum and maximum SV was 26%, which is similar to our percentage error of 27.4%. Chin et al compared the SV derived by a radial artery uncalibrated pulse-contour method (the third generation FloTrac/Vigileo system, Edwards Lifesciences, Irvine, California, USA) with the SV derived by TEE Doppler in patients undergoing laparoscopic prostatectomy.[30] The percentage errors of the absolute values was 53.8%, with a concordance rate of 69.2% (four-quadrant plot), and the mean angular bias 20.6 degrees with radial limits of agreement of ±51.5 degrees in the polar plot. They concluded that the third-generation FloTrac/Vigileo system was not reliable in measuring SV or in tracking changes in the SV after fluid administration in the high systemic vascular resistance state. Recently, a novel CO monitoring, esCCO (Nihon Kohden, Tokyo, Japan), became available for clinical practice and the accuracy of esCCO has been evaluated thoroughly.[8][9][20][31][32] The principle of esCCO estimation is based on the assessment of pulse wave transit time after first calibrating it using patient specific parameters including height, weight, gender, and age as well as heart rate and pulse pressure. Biais et al compared the ability of esCCO with CO measured by transthoracic echocardiography to track cardiovascular responses in critically ill patients.[32] The percentage error was 61% before and 59% after therapeutic interventions. The concordance rate of the four-quadrant plot was 84%, and polar plot analysis revealed a mean angular bias (SD) of −11 (24) degrees with a radial limits of agreement of ± 50 degrees and angular concordance rate of 70%. Their results are comparable with a study by Bataille et al, who found a percentage error of 49%, a concordance rate (four-quadrant analysis) of 73%, and a mean angular bias of -9 degrees with radial limits of agreement of ± 45 degrees and angular concordance rate of 82% in the polar plot analysis.[20] Our results (percentage error of 27%, the concordance rate of 85% in four-quadrant plot analysis, a mean angular bias of 2 degrees with a radial limits of agreement of ±41 degrees and angular concordance rate of 86% in the polar plot analysis) appear superior to the previously published results. This may be attributable to the homogeneity of our patient population or the difference in the formula for estimating SV. The esCCO derives SV proportionally, depending on the time interval from the peak of the R-wave of the electrocardiogram to the upstroke of the pulse oximetry waveform. The esCCO then estimates SV by linear approximation and hence does not provide reliable measurements when changes in arterial compliance or peripheral resistance occur.[20] We estimated SV from PWV using Bramwell-Hill equation. Our method does not need calibration but it requires knowledge of the patients height, weight, gender, and measurements of pulse wave velocity and pulse pressure.

There are several limitations to our study. The greatest downsides of our technique are inaccuracies in estimating the vascular path length and arterial tree blood volume. Vascular path length was calculated from height, age and gender, however, we did not measure it directly in each patient. The arterial tree blood volume was estimated from the predicted total circulating blood volume (TBV) as we could not measure it directly. These inaccuracies do not affect individual tracking of changes in SV under constant TBV, though they affect overall agreement and might be pronounced in states of hemodynamic disturbances. (e.g. acute blood loss). Nevertheless, we have previously published the relationship between SV, HR, BP, and PWV and found that PWV was negatively correlated with SV at the level of the single heartbeat.[33] This suggests that the changes in PWV and PWV based SV are able to capture the changes in SV at the level of single heartbeat. Using a pig model, Kamoi et al. demonstrated that PWV variability can accurately capture the changes in SV if the main cause of fluctuations in SV resulted from changes in preload.[34] This suggests that PWV based SV estimation might be reliable even in states of acute blood loss. Clearly further studies are needed to validate the performance of the present method after volume infusion or in the setting of acute hemorrhage. Further methodological limitations are that the Bramwell-Hill equation is under modeling assumptions such that the vessel wall thickness is small compared to the diameter and that the circulating fluid within the vessel is incompressible and nonviscous.[35] In addition, the simplification of the Bramwell-Hill equation using dV and dP extrapolation to SV and PP is accurate only in patients with no or only minor amplifications of PP from the aorta to the periphery.[12] Since we used aortic to radial PWV to calculate SV, these assumptions may affect the accuracy of our PWV based SV estimation. Also, our study subjects represent a small and homogenous group of overweight and primarily male patients with cardiovascular diseases, who were studied under relatively stable hemodynamic conditions. It is unclear how this method would work in any other setting, or patient cohort.

Another limitation is that we used TEE instead of a PAC as our reference method. As such we cannot extrapolate the agreement and trending ability of our test method with the thermodilution technique. It should also be noted that we performed all measurements manually and hence our results dependent on individual observer judgment. However, two independent readers extracted data and inter-observer variability of SV_PWV_ was acceptable. Moreover, there are timing and bandwidth issues that arise when comparing disparate types of waveforms (flow and pressure) and there is always the potential for hardware-related temporal lags on the two data streams (flow and pressure), which we did not account for in our study. Lastly, a potential for a hardware related confounder is the presence of bubbles in the manometer tubing which could well have damped the signal and distorted the waveform.

## Conclusions

SV estimated from PWV was clinically acceptable and interchangeable with SV measured by TEE. Although the trending ability didn’t reach the defined acceptable range, our PWV based SV/ CO monitor shows clinical promise since its real time, non-invasive and continuous. Further studies in patient population with rapid changes in volume status such as hemorrhage are required to investigate the clinical utility of the proposed methodology.

## References

[1] Cannesson M, Pestel G, Ricks C, Hoeft A, Perel A. Hemodynamic monitoring and management in patients undergoing high risk surgery: a survey among North American and European anesthesiologists. Crit Care. BioMed Central Ltd; 2011;15: R197 10.1186/cc10364 21843353 PMC3387639

[2] Marik PE. Obituary: pulmonary artery catheter 1970 to 2013. Ann Intensive Care. 2013;3: 38 10.1186/2110-5820-3-38 24286266 PMC4175482

[3] National Heart, Lung, and Blood Institute Acute Respiratory Distress Syndrome (ARDS) Clinical Trials Network, Wheeler AP, Bernard GR, Thompson BT, Schoenfeld D, Wiedemann HP, et al Pulmonary-artery versus central venous catheter to guide treatment of acute lung injury. N Engl J Med. 2006; 354: 2213–24. 10.1056/NEJMoa061895 16714768

[4] Peyton PJ, Chong SW. Minimally Invasive Measurement of Cardiac Output during Surgery and Critical Care. 2010; 113: 1220–35. 10.1097/ALN.0b013e3181ee3130 20881596

[5] Perrino a C, Harris SN, Luther M a. Intraoperative determination of cardiac output using multiplane transesophageal echocardiography: a comparison to thermodilution. Anesthesiology. 1998 pp. 350–357. 9710392 10.1097/00000542-199808000-00010

[6] Harris SN, Luther MA, Perrino A.C. J. Multiplane transesophageal echocardiographic acquisition of ascending aortic flow velocities: A comparison with established techniques. J Am Soc Echocardiogr. 1999;12: 754–760. 10477420 10.1016/s0894-7317(99)70026-x

[7] Mehta Y, Arora D. Newer methods of cardiac output monitoring. World J Cardiol. 2014;6: 1022–9. 10.4330/wjc.v6.i9.1022 25276302 PMC4176793

[8] Smetkin AA, Hussain A, Fot E V., Zakharov VI, Izotova NN, Yudina AS, et al Estimated continuous cardiac output based on pulse wave transit time in off-pump coronary artery bypass grafting: a comparison with transpulmonary thermodilution. J Clin Monit Comput. Springer Netherlands; 2016; 1–10. 26951494 10.1007/s10877-016-9853-5

[9] Ishihara H, Sugo Y, Tsutsui M, Yamada T, Sato T, Akazawa T, et al The ability of a new continuous cardiac output monitor to measure trends in cardiac output following implementation of a patient information calibration and an automated exclusion algorithm. J Clin Monit Comput. 2012;26: 465–471. 10.1007/s10877-012-9384-7 22854918 PMC3494869

[10] Barodka VM, Joshi BL, Berkowitz DE, Hogue CW, Nyhan D. Implications of vascular aging. Anesth Analg. 2011;112: 1048–1060. 10.1213/ANE.0b013e3182147e3c 21474663 PMC3694586

[11] Bramwell JC, Hill A V. The Velocity of the Pulse Wave in Man. Proc R Soc London B Biol Sci. 1922;93.

[12] Chemla D, Hébert JL, Coirault C, Zamani K, Suard I, Colin P, et al Total arterial compliance estimated by stroke volume-to-aortic pulse pressure ratio in humans. Am J Physiol. 1998;274: H500–5. Available: http://www.ncbi.nlm.nih.gov/pubmed/9486253 9486253 10.1152/ajpheart.1998.274.2.H500

[13] Nichols W, O’Rourke M, Vlachopoulos C. Mcdonaldʼs Blood Flow in Arteries. Shock. 1998;9: 456.

[14] Nadler SB, Hidalgo JH, Bloch T. Prediction of blood volume in normal human adults. Surgery. 1962;51: 224–32. 21936146

[15] Pai MP, Paloucek FP. The Origin of the “Ideal” Body Weight Equations. Ann Pharmacother 2000;34: 1066–9. 10981254 10.1345/aph.19381

[16] Erstad BL. Dosing of medications in morbidly obese patients in the intensive care unit setting. 2004;30: 18–32. 10.1007/s00134-003-2059-6 14625670

[17] Thongprayoon C, Cheungpasitporn W, Akhoundi A, Ahmed AH, Kashani KB. Actual versus ideal body weight for acute kidney injury diagnosis and classification in critically Ill patients. BMC Nephrol 2014; 15:176 10.1186/1471-2369-15-176 25398596 PMC4236495

[18] Hirani V, Aresu M. Development of new demi-span equations from a nationally representative sample of older people to estimate adult height. J Am Geriatr Soc. 2012;60: 550–554. 10.1111/j.1532-5415.2011.03832.x 22315968

[19] Jones B, Jarvis P, Lewis J a, Ebbutt a F. Trials to assess equivalence: the importance of rigorous methods. BMJ. 1996;313: 36–39. 8664772 10.1136/bmj.313.7048.36 PMC2351444

[20] Bataille B, Bertuit M, Mora M, Mazerolles M, Cocquet P, Masson B, et al Comparison of esCCO and transthoracic echocardiography for non-invasive measurement of cardiac output intensive care. Br J Anaesth. 2012;109: 879–886. 10.1093/bja/aes298 22907340

[21] Critchley LA, Critchley JA. A Meta-Analysis of Studies Using Bias and Precision Statistics to Compare Cardiac Output Measurement Techniques. J Clin Monit Comput 1999; 15: 85–91. 12578081 10.1023/a:1009982611386

[22] Critchley LA, Lee A, Ho AMH. A critical review of the ability of continuous cardiac output monitors to measure trends in cardiac output. Anesth Analg. 2010;111: 1180–1192. 10.1213/ANE.0b013e3181f08a5b 20736431

[23] Critchley LA, Yang XX, Lee A. Assessment of trending ability of cardiac output monitors by polar plot methodology. J Cardiothorac Vasc Anesth. Elsevier Inc.; 2011;25: 536–546. 10.1053/j.jvca.2011.01.003 21419654

[24] Cecconi M, Rhodes A, Poloniecki J, Rocca G Della, Grounds RM. Bench-to-bedside review: The importance of the precision of the reference technique in method comparison studies–with specific reference to the measurement of cardiac output. Crit Care. 2009;13: 201 10.1186/cc7129 19183431 PMC2688094

[25] Robson SC, Murray A, Peart I, Heads A, Hunter S. Reproducibility of cardiac output measurement by cross sectional and Doppler echocardiography. Br Heart J. 1988;59: 680–4. 3395526 10.1136/hrt.59.6.680 PMC1276875

[26] Linton NWF, Linton RAF. Is comparison of changes in cardiac output, assessed by different methods, better than only comparing cardiac output to the reference method? Br J Anaesth. 2002;89: 336-337-339. 10.1093/bja/aef530 12378677

[27] Saugel B, Grothe O, Wagner JY. Tracking changes in cardiac output: Statistical considerations on the 4-quadrant plot and the polar plot methodology. Anesth Analg. 2015;121: 514–524. 10.1213/ANE.0000000000000725 26039419

[28] Liu AB, Hsu PC, Chen ZL, Wu HT. Measuring pulse wave velocity using ECG and photoplethysmography. J Med Syst. 2011;35: 771–777. 10.1007/s10916-010-9469-0 20703725

[29] Castro V De, Lhotel L, Mabrouk N, Perel A, Coriat P. Comparison of stroke volume (SV) and stroke volume respiratory variation (SVV) measured by the axillary artery pulse-contour method and by aortic Doppler echocardiography in patients undergoing aortic surgery. 2006;97: 605–610. 10.1093/bja/ael236 17012308

[30] Chin JH, Kim WJ, Choi JH, Han YA, Kim SO, Choi WJ. Unreliable tracking ability of the third-generation FloTrac/Vigileo^TM^ system for changes in stroke volume after fluid administration in patients with high systemic vascular resistance during laparoscopic surgery. PLoS One. 2015;10: e0142125 10.1371/journal.pone.0142125 26529592 PMC4631474

[31] Sugo Y, Ukawa T, Takeda S, Ishihara H, Kazama T, Takeda J. A novel continuous cardiac output monitor based on pulse wave transit time. 2010 Annu Int Conf IEEE Eng Med Biol Soc EMBC’10. 2010; 2853–2856. 10.1109/IEMBS.2010.5626343 21095971

[32] Biais M, Berthez??ne R, Petit L, Cottenceau V, Sztark F. Ability of esCCO to track changes in cardiac output. Br J Anaesth. 2015;115: 403–410. 10.1093/bja/aev219 26209443

[33] Obata Y, Mizogami M, Singh S, Nyhan D, Berkowitz DE, Steppan J, et al. Clinical Study The Effects of Hemodynamic Changes on Pulse Wave Velocity in Cardiothoracic Surgical Patients. 10.1155/2016/9640457 PMC5120184 27900333

[34] Kamoi S, Pretty C, Chiew YS, Davidson S, Pironet A, Desaive T, et al Relationship between Stroke Volume and Pulse Wave Velocity. IFAC-PapersOnLine. 2015;48: 285–290.

[35] Westenberg JJM, van Poelgeest EP, Steendijk P, Grotenhuis HB, Jukema JW, de Roos A. Bramwell-Hill modeling for local aortic pulse wave velocity estimation: a validation study with velocity-encoded cardiovascular magnetic resonance and invasive pressure assessment. J Cardiovasc Magn Reson. BioMed Central Ltd; 2012;14: 2 10.1186/1532-429X-14-2 22230116 PMC3312851

